# Distinct pattern of matrix metalloproteinase 9 and tissue inhibitor of metalloproteinase 1 mRNA expression in human colorectal cancer and liver metastases.

**DOI:** 10.1038/bjc.1995.376

**Published:** 1995-09

**Authors:** Z. S. Zeng, J. G. Guillem

**Affiliations:** Department of Surgery, Memorial Sloan-Kettering Cancer Center, New York, New York 10021, USA.

## Abstract

**Images:**


British Journal of Cancer (1995) 72, 575-582

© 1995 Stockton Press All rights reserved 0007-0920/95 $12.00

### Distinct pattern of matrix metalloproteinase 9 and tissue inhibitor of metalloproteinase 1 mRNA expression in human colorectal cancer and liver metastases

ZS Zeng and JG Guillem

*Colorectal Service, Department of Surgery, Memorial Sloan-Kettering Cancer Center, 1275 York Avenue, New York, New York 10021, USA.*

**Summary** The matrix metalloproteinases (MMPs) and tissue inhibitors of metalloproteinases (TIMPs) are perceived as essential for tumour invasion and metastasis. In the present study, we compare the topographical pattern of MMP-9 and TIMP-1 expression in colorectal cancer and liver metastases by *in situ* hybridisation. TIMP-1 mRNA was detected in all 26 colorectal cancers examined, while only 18 out of 26 (69.2%) were positive for MMP-9. Both MMP-9 and TIMP-1 mRNA were observed in all ten liver metastases but were absent in three adenomas and in all normal colonic mucosa and liver. There was no association between MMP-9 or TIMP-1 mRNA expression and degree of differentiation or size of tumours. MMP-9 and TIMP-1 mRNA were similarly observed in the peritumour stroma cells rather than in tumour cells themselves. MMP-9 mRNA-positive cells were round and identified as macrophages by immunostaining with an anti-macrophage antibody (KP1), while TIMP-1 mRNA was detected in spindle-shaped stromal cells. In liver metastases, MMP-9 localised within peritumour stroma or at the interface between the tumour stroma and normal liver, whereas TIMP-1 mRNA was located throughout the malignant tumour stroma. Our data demonstrate a distinct pattern of MMP-9 and TIMP-1 mRNA expression in colorectal cancer and liver metastases suggesting distinct cellular origins as well as separate patterns of regulation.

**Keywords:** *in situ* hybridisation; matrix metalloproteinase; tissue inhibitor of metalloproteinase; type IV collagenase

Matrix metalloproteinases (MMPs) are a family of metal-dependent endopeptidases with proteolytic activities for various components of the extracellular matrix (Liotta and Stetler-Stevenson, 1991; Ennis and Matrisian, 1994). The MMP family comprises at least nine members (Woessner, 1994). All family members are secreted in a latent form that becomes active upon cleavage of a 10 000 dalton peptide (Ennis and Matrisian, 1994). Since type IV collagen is a major component of basement membranes, the 72 kDa (MMP-2) and 92 kDa (MMP-9) type IV collagenases are of particular interest.

Over the last decade, much attention has focused on the role of MMPs in tumour invasion and metastases (Liotta and Stetler-Stevenson, 1991; Murphy *et al.*, 1989). Both *in vitro* and *in vivo* data have indicated that MMPs participate, via accelerated breakdown of extracellular matrices, in tumour invasion and metastases (Liotta *et al.*, 1980; Nakajima *et al.*, 1987; Ura *et al.*, 1989). Furthermore, increased production of MMP has been associated with increased invasive and metastatic potential in several human malignant tumours (Levy *et al.*, 1991; Urbanski *et al.*, 1992; Boag and Young, 1993; Brown *et al.*, 1993). Although, MMP-9 is secreted by a wide range of cells, including macrophages, neutrophils, capillary endothelial cells and trophoblasts, from both normal and malignant tissue (Hibbs *et al.*, 1985, 1987; Herron *et al.*, 1988; Werb and Alexander, 1993) in primary colorectal cancer, it appears that MMP-9 mRNA may be macrophage in origin (Pyke *et al.*, 1993).

MMP activity, *in vivo*, is thought to be regulated in part by natural tissue inhibitor proteins such as tissue inhibitors of metalloproteinases (TIMP) (Liotta and Stetler-Stevenson, 1991; Ennis and Matrisian, 1994). Thus far, three TIMP genes (TIMP-1, TIMP-2 and TIMP-3) have been identified (Murphy *et al.*, 1981; Stetler-Stevenson *et al.*, 1989; Pavloff *et al.*, 1992). TIMP-1 is a glycoprotein with an apparent molecular size of 28.5 kDa and is produced by a variety of human tissues and many human tumour cell lines (Murphy *et al.*, 1981; Stricklin and Welgus, 1983; Dean *et al.*, 1992; Sato *et al.*, 1992). TIMP-1 inhibits MMPs by forming a 1:1 complex with activated MMP-1, MMP-3 and MMP-9 (Liotta and Stetler-Stevenson, 1991; Ennis and Matrisian., 1994). Although several studies have shown an inverse correlation between TIMP-1 levels and the metastatic potential of murine and human tumour cells (Schultz *et al.*, 1988; Khokha *et al.*, 1989; Alvarez *et al.*, 1990), we and others have demonstrated a direct correlation between TIMP-1 expression and clinical aggressiveness of colorectal cancer (Guillem *et al.*, 1990; Lu *et al.*, 1991; Zeng *et al.*, 1995). Recently, using Northern blot analysis of whole-tissue homogenates, we demonstrated a parallel overexpression of TIMP-1 and MMP-9 mRNA in colorectal cancer and liver metastases, suggesting co-regulation of these two genes (Zeng *et al.*, 1993). However, because of tissue cellular heterogeneity, the exact cellular origin of TIMP-1 and MMP-9 mRNA in colorectal cancer and liver metastases homogenates remained unclear. In the present study, *in situ* hybridisation analysis was used to investigate the cellular distribution of TIMP-1 and MMP-9 mRNA expression in human colorectal cancer and liver metastases.

### Materials and methods

### Tissue samples

All surgical samples used in this study were randomly obtained from the operating room immediately after resection. Eighteen primary colorectal cancers and corresponding normal mucosa and seven colorectal cancer liver metastases and matched normal liver were examined by Northern blot analysis. In addition, 26 primary colorectal cancers, three benign adenomas and ten liver metastases as well as corresponding normal adjacent tissue were examined by *in situ* hybridisation. Table I summarises the clinicopathological variables of these 26 primary colorectal cancers. The Surgical Pathological Laboratory of the Memorial Sloan-

Correspondence: JG Guillem, Colorectal Service, MSKCC, 1275 York Avenue, NY 10021, USA
Received 19 January 1995; revised 19 April 1995; accepted 3 May 1995

**Table I** Clinicopathological characteristics of 26 colorectal cancers and *in situ* MMP-9 and TIMP-1 hybridisation status

| *Case* | *Age* | *Sex* | *Location*[a] | *Diff*[b] | *Size (cm) (Max)* | *Dukes' stage* | *In Situ hybridisation* TIMP-1 | MMP-9 |
|---|---|---|---|---|---|---|---|---|
| 1 | 80 | F | RE | M | 6.5 | C | + | + |
| 2 | 65 | F | RS | M | 4.0 | A | + | – |
| 3 | 66 | F | R | M | 4.0 | C | + | – |
| 4 | 78 | M | RS | M | 4.8 | D | + | + |
| 5 | 71 | M | RE | M | 3.5 | A | + | – |
| 6 | 54 | M | RE | P | 7.0 | D | + | + |
| 7 | 81 | F | S | P | 3.2 | C | + | + |
| 8 | 55 | F | R | M | 6.0 | B | + | + |
| 9 | 64 | F | S | M | 2.5 | B | + | – |
| 10 | 63 | M | R | M | 5.0 | C | + | + |
| 11 | 72 | F | RE | M | 10.0 | D | + | + |
| 12 | 67 | M | S | M | 4.5 | D | + | + |
| 13 | 64 | M | R | M | 3.0 | A | + | – |
| 14 | 32 | F | RC | M | 4.5 | D | + | + |
| 15 | 77 | M | R | M | 3.2 | B | + | – |
| 16 | 47 | M | S | M | 6.0 | D | + | + |
| 17 | 75 | M | RE | M | 6.5 | B | + | + |
| 18 | 78 | M | RE | M | 7.5 | C | + | + |
| 19 | 74 | F | R | M | 3.6 | A | + | + |
| 20 | 62 | M | R | P | 10.0 | D | + | + |
| 21 | 62 | F | R | M | 9.0 | D | + | + |
| 22 | 86 | M | R | M | 6.0 | B | + | + |
| 23 | 50 | M | L | M | 3.0 | D | + | + |
| 24 | 69 | F | L | M | 3.7 | C | + | – |
| 25 | 71 | F | R | M | 4.5 | A | + | – |
| 26 | 74 | F | RC | M | 7.0 | C | + | + |

[a]R, right colon; L, left colon; S, sigmoid colon; RS, rectum-sigmoid colon; RE, rectum.
[b]Diff refers to tumour differentiation: M, moderate; P, poor.

Kettering Cancer Center (MSKCC) performed routine histopathological examination on the resected specimens using haematoxylin and eosin staining.

*Tissue processing*

*Northern blot analysis* Specimens obtained from the tumour edge, avoiding a necrotic centre, were quick-frozen in liquid nitrogen and stored at –80°C. Samples were handled and stored under strict RNAse-free conditions. Samples of normal mucosa were obtained from the surgical resection margin by sharply dissecting the mucosa off the muscularis.

In situ *analysis* Specimens were fixed in 4% paraformaldehyde overnight at 4°C, sequentially dehydrated with 50%, 70%, 85%, 95% and 100% ethanol and embedded in paraffin. To prevent RNA degradation, reagents were treated with diethyl pyrocarbonate (0.1%).

*Preparation of RNA probes*

Sense and antisense $^{35}$S-labelled RNA probes were prepared from human TIMP-1 and MMP-9 cDNAs cloned in Bluescript KS (Stratagene). The TIMP-1 sense probe was generated by T3 polymerase following digestion with *Kpn*L, while the antisense probe was generated by T7 polymerase after *Eco*RI template digestion. For MMP-9 RNA probes, sense and antisense plasmid vectors were linearised with *Xba*L and *Eco*RI respectively. Transcribed RNA was labelled with [α-$^{35}$S]UTP (1200 Ci mmol$^{-1}$, Dupont, NEW). DNA template was removed by incubating with RNAse-free DNAse. Ten micrograms of tRNA was added and samples were extracted with phenol–chloroform. RNA probes were hydrolysed with sodium carbonate buffer, pH 10.2, for 60 min at 60°C, neutralised and ethanol precipitated. Probes were redissolved in 10 mM DTT at a final concentration of 1.5 ng μl$^{-1}$ and stored at –20°C.

In situ *hybridisation*

*Prehybridisation* Paraffin sections 5–10 μm thick were dried at 42°C overnight, deparaffinised in xylene and then redehydrated by passage through graded ethanol. Slides were incubated with proteinase K (100 μg ml$^{-1}$) at room temperature for 7.5 min and then serially dipped in PBS, washed in 2 mg ml$^{-1}$ glycine (w/v) and finally washed in freshly prepared triethanolamine buffer containing 0.25% acetic anhydride for 10 min.

*Hybridisation* The labelled probes were diluted in hybridisation buffer containing 50% formamide, 10% dextran sulphate, 0.1 M DTT and Denhardt's. $^{35}$S-labelled RNA probe was added to each slide in a 25 μl volume of hybridisation mixture. The slides were covered with coverslip and incubated at 57°C overnight in a 50% formamide, 2 × SSC humidified chamber.

*Post-hybridisation* After hybridisation, coverslips were gently removed with 4 × SSC and slides washed at room temperature in 4 × SSC for 1 h and then in 2 × SSC, 50% formamide, 10 mM DTT solution for 40 min at 68°C. Slides were then treated with 20 μg ml$^{-1}$ RNAse A in 20 mM Tris, pH 7.5, 0.5 M sodium chloride, 1 mM EDTA at 37°C for 30 min followed by washing in the same buffer without RNAse A for 30 min. The final washes were in 2 × SSC, 50% formamide, 1 mM DTT for 40 min at 68°C, 2 × SSC for 5 min at room temperature and 0.1 × SSC for 15 min at 50°C. Slides were dehydrated through graded ethanol and air dried.

*Autoradiography* Autoradiographic detection of the hybrids was carried out by dipping in Kodak NTB-2 emulsion (Eastman Kodak, Rochester, NY, USA) at 42°C under safe light and dried at room temperature for at least 2 h. Slides were placed in a light tight box at 4°C for 1–2 weeks. After exposure, slides were subsequently developed in Kodak D-19 developer for 4 min at 15°C, washed in water and fixed in Kodak fixer. Tissue sections were counterstained with haematoxylin and eosin (H&E) and mounted. Silver grains were visualised by dark-field microscopy (Nikon).

*Northern blot hybridisation*

RNA was extracted by the guanidium isocyanide/caesium chloride method as previously described (Zeng *et al.*, 1994*a*). Ten micrograms of total RNA was electrophoresed on 1.0% agarose–formaldehyde gel and blotted onto a Duralon-UV

membrane (Stratagene). TIMP-1 and MMP-9 DNA probes were radiolabelled with [$^{32}$P]dCTP by the random primer technique (Feinberg and Vogelstein, 1983). A 28S oligo probe was used as an internal control (Barbu and Dautry, 1989) for equal RNA loading and ethidium bromide staining of gels to confirm equal RNA transfer. Hybridised RNA was quantitated by LKB XL laser densitometry (Pharmacia LKB Biotechnology, Uppsala, Sweden). The results were expressed as a ratio of the average fold increase of transcript in tumours to that in the paired normal tissues. The difference in standardised TIMP-1 and MMP-9 between tumour and paired normal tissue was assessed by the paired $t$-test. Significant differences were analysed by Student's $t$-test.

*Immunohistochemistry*

Tissue sections adjacent to those used for *in situ* hybridisation were used for immunohistochemistry. Tissue macrophages were identified by peroxidase-antiperoxidase immunostaining using a monoclonal anti-macrophage antibody KP-1 (CD-68). This antibody is known to react specifically with tissue macrophages (Pulford *et al.*, 1989). The immunohistochemical staining technique was performed as previously described (Zeng *et al.*, 1994*b*).

### Results

*Expression of MMP-9 and TIMP-1 in colorectal cancer and liver metastases*

Northern blot analysis of both MMP-9 and TIMP-1 in 18 primary colorectal cancers and matched adjacent normal mucosa as well as seven colorectal cancer liver metastases and matched normal liver revealed a parallel overexpression of MMP-9 and TIMP-1 mRNA in both human colorectal cancer and liver metastases (Figure 1). The mean tumour/normal (T/N) fold increases of TIMP-1 and MMP-9 in primary cancer were $9.1 \pm 1.9$ ($P<0.01$) and $12.9 \pm 5.0$ ($P<0.01$), while in liver metastases they were $11.1 \pm 3.6$ ($P<0.05$) and $3.47 \pm 1.2$ ($P<0.05$) respectively.

In situ *hybridisation of MMP-9 and TIMP-1 mRNA in primary colorectal cancer*

As seen in Figure 2, primary colorectal cancers show detectable signals for MMP-9 and TIMP-1 mRNA. All 26 colorectal cancers were positive for TIMP-1 expression, while only 18 out of 26 (69.2%) colorectal cancers were positive for MMP-9 (Table I). Intensity of staining varied between different cases and in different areas of the same tumour. In three adenomas and in corresponding normal mucosa, insignificant (background) quantities of MMP-9 and TIMP-1 mRNA were detected and the control sense probes for MMP-9 and TIMP-1 showed only background autographic signals (figures not shown). Since silver grain signals occurred only with the antisense probe and none were detected with the sense probe, the hybridisation was felt to be specific.

The correlation between MMP-9 and TIMP-1 mRNA expression in 26 colorectal cancer specimens and the relationship to clinicopathological variables is summarised in Table I. MMP-9 and TIMP-1 mRNA expression did not correlate with either the degree of tumour differentiation or the size of the tumours. Figure 3 shows MMP-9 mRNA expression in relation to Dukes' stage. MMP-9 mRNA expression progressively increased with advancing stages (Dukes' A to 'Dukes' D').

In situ *hybridisation of MMP-9 and TIMP-1 mRNA in colorectal cancer liver metastases*

*In situ* hybridisation analysis revealed a remarkably specific signal for MMP-9 (Figure 4) and TIMP-1 (Figure 5) mRNA transcripts in all ten liver metastases from colorectal cancer. No expression was detected in adjacent normal liver and hybridisations with MMP-9 and TIMP-1 sense probes were also negative (data not shown).

*Distinct localisation of MMP-9 and TIMP-1 expression*

Although both MMP-9 and TIMP-1 mRNA signals were strongest within stromal cells of tumours, the spatial distributions of MMP-9 and TIMP-1 mRNA were distinctly different. In order to compare directly the spatial expression of MMP-9 and TIMP-1, we hybridised serial sections of the same samples. As seen in serial sections (Figure 2), MMP-9 mRNA hybridisation resulted in a more focused signal, strongest within a small population of stromal cells encircling the tumour epithelial cells (Figure 2b and c), whereas TIMP-1 mRNA hybridisation produced a diffuse scattering signal (Figure 2e and f).

The general expression patterns of MMP-9 and TIMP-1 mRNA in liver metastases were similar to those in primary colorectal cancers. Comparison of MMP-9 and TIMP-1 mRNA expression in liver metastases indicates that, although hybridisation signals for MMP-9 and TIMP-1 transcripts were clearly localised in tumour stroma, MMP-9 and TIMP-1 mRNA have distinctly different patterns of distribution. As seen in Figure 4a and b, large numbers of MMP-9-positive cells were localised at the interface between the liver metastases lesion (LM) and surrounding normal liver parenchyma (NL). However, TIMP-1 mRNA was found throughout malignant tumour stroma (Figure 5a and b).

Higher magnifications of the area bordered by arrows in Figure 2b and e are shown in Figure 2c and f. The MMP-9 mRNA signals were seen in a small population of stromal cells encircling the tumour epithelial cells (Figure 2c), whereas TIMP-1-labelled cells are clearly spindle-shaped stromal cells (Figure 2f). When seen in liver metastases under higher magnification, MMP-9 mRNA-positive stroma cells (Figure 4b, arrow marked) have a circular pattern (Figure 4c), while TIMP-1-positive cells (Figure 5b, arrow marked) are spindle like in shape (Figure 5c).

To identify the cells expressing MMP-9 mRNA three colorectal cancer and two liver metastases samples were examined by immunohistochemical staining with the monoclonal antibody KP1 (CD68), known to be macrophage specific (Pulford *et al.*, 1989). Figure 6 shows that cells

**Figure 1** MMP-9 and TIMP-1 expression in colorectal cancer and liver metastases from colorectal cancer. MMP-9 and TIMP-1 cDNA probe co-hybridisation was performed from tumour and adjacent normal tissue (T, tumour; N, normal mucosa; M, liver metastases; L, normal liver tissue). Blots were subsequently stripped and reprobed with the 28S probe.

**Figure 2** Microphotographs showing *in situ* hybridisation for TIMP-1 and MMP-9 in serial sections of primary human colon cancer. Bright-field and corresponding dark-field microphotography of MMP-9 mRNA (**a**, **b** and **c**) and TIMP-1 mRNA (**d**, **e** and **f**) detected by *in situ* hybridisation with $^{35}$S-labelled antisense RNA probe. (**a** and **d**) Low-power dark-field view of colon cancer. (**b**, and **e**) Light-field view of the same areas of **a** and **d**. Both TIMP-1 and MMP-9 mRNA are located in malignant tumour stroma; no signal is observed in malignant epithelium. Higher magnifications of the area bordered by arrows in **b** and **e** are shown in **c** and **f**. MMP-9 mRNA was found in a circular pattern of stromal cells (**c**), while TIMP-1 mRNA localised primarily in fibroblast-like stromal cells (**f**).

**Figure 3** MMP-9 mRNA expression (■, positive; □, negative) in primary human colorectal cancer in relation to Dukes' stage.

positive for MMP-9 mRNA expression in liver metastases have a circular morphology (Figure 6a) and were identified as macrophages by immunostaining of adjacent sections with anti-macrophage antibody (Figure 6b). As noted, not all macrophages were positive for MMP-9 mRNA expression.

### Discussion

Our present *in situ* hybridisation results are consistent with our prior Northern blot demonstration of MMP-9 and TIMP-1 mRNA overexpression in primary colorectal cancer and liver metastases (Zeng *et al.*, 1993). Our striking novel observation is the distinct spatial expression of MMP-9 and TIMP-1 mRNA within pericancer stroma in both primary colorectal cancer and liver metastases. Our findings provide

**Figure 4** MMP-9 mRNA expression in liver metastases from colorectal cancer. Sections were hybridised with a $^{35}$S-labelled antisense RNA probe specific for MMP-9 mRNA. Dark-field (**a**) and bright-field (**b**) microphotographs demonstrate MMP-9 mRNA expression limited to the interface between liver metastases lesion (LM) and normal liver (NL). Higher magnification of the area bordered by arrows in **b** suggests that MMP-9 mRNA-positive stroma cells have a circular morphology (**c**).

**Figure 5** TIMP-1 mRNA expression in liver metastases from colorectal cancer. Sections were hybridised with a $^{35}$S-labelled antisense RNA probe specific for TIMP-1 mRNA. Dark-field (**a**) and bright-field (**b**) microphotographs demonstrate TIMP-1 signals principally in malignant tumour stroma. No signal is observed in malignant epithelium. Higher magnification of the area bordered by arrows in **b** reveals TIMP-1 mRNA to be located in fibroblastlike stromal cells (**c**).

the evidence that MMP-9 and TIMP-1 mRNA are predominantly stromal in origin in both primary colorectal cancer and liver metastases. To our knowledge, this is the first report demonstrating a distinct spatial expression of MMP-9 and TIMP-1 mRNA in liver metastases from colorectal cancers.

MMP-9 is produced *in vitro* by a variety of cell types, including fibroblasts, endothelial cells, keratinocytes, macrophages and chondrocytes (Werb and Alexander, 1993). The synthesis of MMP-9 has been induced by SV-40 transformation of human fibroblasts (Wilhelm *et al.*, 1989). Under normal circumstances this enzyme is a major secretion product of tissue macrophages (Wilhelm *et al.*, 1989) and has been found in the granules of polymorphonuclear leucocytes and in cultured keratinocytes (Wilhelm *et al.*,1989). In human granuloma annulare and necrobiosis lipoidica diabeticorum, MMP-9 is expressed in eosinophils (Saarialho-Kere *et al.*, 1993). Our observation that MMP-9 mRNA may be produced by peritumour macrophages is consistent with a study (Pyke *et al.*, 1993) in which MMP-9 was found in stroma cells of colorectal cancers. Similar to colon cancers, *in situ* hybridisation studies have shown MMP-9 mRNA to be localised in the stroma of breast (Davies *et al.*, 1993*a*) and bladder cancers (Davies *et al.*, 1993*b*). In human skin cancer, six of nine squamous cell carcinomas expressed MMP-9 mRNA (Pyke *et al.*, 1992). However, none of the basal cell carcinomas, a rarely metastatic tumour, expressed MMP-9 mRNA (Pyke *et al.*, 1992).

The role for several metalloproteinases in colorectal tumorigenesis is supported by Newell *et al.* (1994) who demonstrated matrilysin (MMP-7) expression in both benign and malignant colorectal tumour. However, the expression of stromelysin-1 (MMP-3), stromelysin-3 (MMP-11) and MMP-2 was noted only in colorectal cancer. These findings suggest that MMP-7 expression is an early event and that MMP-3, MMP-11 and MMP-2 expression is

**Figure 6** Identification of MMP-9 mRNA-positive cells. Serial sections of a liver metastases were hybridised with a $^{35}S$-labelled antisense RNA probe specific for MMP-9 mRNA or stained with a macrophage-specific monoclonal antibody KP1 (CD68). (**a**) *In situ* hybridisation. Cells positive for MMP-9 mRNA have a macrophage-like morphology. (**b**) Immunohistochemical staining. MMP-9 mRNA-positive cells were identified as macrophages by immunostaining of adjacent sections. As noted, not all macrophages are positive for MMP-9 mRNA expression.

primarily a late event in colorectal tumorigenesis. The focal expression of MMP-9 at the interface between liver metastases and normal liver and its apparent macrophage origin reported here suggest an important role for macrophages in degrading the extracellular matrix of colorectal cancer liver metastases.

TIMP-1 is expressed *in vitro* by numerous cell types, including fibroblasts (Stricklin and Welgus, 1983), chondrocytes (Gavrilovic *et al.*, 1987) and endothelial (Herron *et al.*, 1988), and vascular smooth muscle cells (DeClerck, 1988). Analysis of human colorectal cancer specimen homogenates have demonstrated an overexpression of both TIMP-1 mRNA and protein when compared with normal colonic mucosa (Guillem *et al.*, 1990; Lu *et al.*, 1991; Zeng *et al.*, 1995). In this study, we have found that, in human colorectal cancer, TIMP-1 mRNA is located in fibroblast-like stromal cells. A previous study on human granuloma annulare and necrobiosis lipoidica diabeticorum demonstrated TIMP-1 mRNA expression in spindle-shaped cells (Saarialho-Kere *et al.*, 1993), suggesting that fibroblasts may serve as a source for TIMP-1. Our *in situ* hybridisation data are consistent with a study by Hewitt *et al.* (1991) which demonstrated increased protein expression of collagenase and TIMP-1 in colorectal cancers compared with adenomas and normal mucosa. The strongest immunohistochemical collagenase and TIMP signals were noted close to islands of neoplastic cells. Furthermore, in this study, the staining intensity in the invasive edge increased for collagenase but decreased for TIMP (Hewitt *et al.*, 1991).

Parallel overexpression of TIMP-1 and MMP-9 mRNA noted in primary colorectal cancer and liver metastases homogenates (Zeng *et al.*, 1993) suggests possible co-regulation of these two important genes *in vivo*. Although the expression of several MMPs (MMP-2,-3,-7,-9 and -11) is elevated in colorectal cancer (Newell *et al.*, 1994), the distinct pattern of cellular TIMP-1 and MMP-9 expression noted by *in situ* hybridisation suggests that elevated TIMP-1 expression may not be simply a response to local increases in MMP-9 expression. This notion is supported by the fact that TIMP-1 has growth-promoting properties (Gasson *et al.*, 1985; Avalos *et al.*, 1988; Bertaux *et al.*, 1991). Hayakawa *et al.* (1992) have found that TIMP-1 accounts for a significant portion of the growth factor activity of serum and is capable of stimulating a wide range of human and bovine cell lines, including those derived from tumours (human breast adenocarcinoma, erythroleukaemia, myelogenous leukaemia and Burkitt's lymphoma). These data, along with the recent observation that TIMP-1 stimulates the secretion of collagenase from human skin fibroblasts (Clark *et al.*, 1994), suggest that, in addition to its role as a metalloproteinase inhibitor, TIMP-1 may also function as a growth factor in the pathogenesis of a variety of diseases. The mechanism of TIMPs' multiple function is presently unknown. However, it is postulated that the domains responsible for growth factor activity are physically distinct from those responsible for regulating metalloproteinase activity (Docherty *et al.*, 1992; Stetler-Stevenson *et al.*, 1992).

In conclusion *in situ* hybridisation analysis has shown distinct patterns of TIMP-1 and MMP-9 mRNA expression in both primary colorectal cancer and liver metastases. Our results demonstrate that MMP-9 and TIMP-1 mRNA are produced not by colorectal cancer cells themselves, but rather by surrounding macrophages and fibroblast-like cells respectively. In addition, the distinct, non-juxtaposed pattern of MMP-9 and TIMP-1 expression suggests that TIMP-1 may not be produced simply in response to local elevations of MMP-9 and suggests that *in vivo* these genes are independently regulated. Further studies are needed to define the colorectal cancer–stroma cell interactions involved in the regulation of MMPs and TIMP productions.

**Acknowledgements**
JG Guillem is supported, in part, by a Career Development Award from the American Cancer Society and the Giovanna Sbarro Cancer Research Foundation. ZS Zeng is recipient of the Stuart HQ Quan MD Research Fellowship. We thank Dr Stetler-Stevenson of the NCI for making available the TIMP-1 and MMP-9 riboprobe vectors.

**References**

ALVAREZ OA, CARMICHAEL DF AND DECLERCK YA. (1990). Inhibition of collagenolytic activity and metastasis of tumor cells by a recombinant human tissue inhibitor of metalloproteinases. *J. Natl. Cancer Inst.*, **82**, 589–595.

AVALOS BR, KAUFMAN SE, TOMONAGA M, WILLIAMS RE, GOLDE DW AND GASSON JC. (1988). K562 cells produce and respond to human erythroid-potentiating activity. *Blood*, **71**, 1720–1725.

BARBU V AND DAUTRY F. (1989). Northern blot normalization with a 28S rRNA oligonucleotide probe. *Nucleic Acids. Res.*, **17**, 7115.

BERTAUX B, HORNEBECK W, EISEN AZ AND DUBERTRET L. (1991). Growth stimulation of human keratinocytes by tissue inhibitor of metalloproteinases. *J. Invest. Dermatol.*, **97**, 679–685.

BOAG AH AND YOUNG ID. (1993). Immunohistochemical analysis of type IV collagenase expression in prostatic hyperplasia and adenocarcinoma. *Mod. Pathol.*, **6**, 65–68.

BROWN PD, BLOXIDGE RE, STUART NSA, GATTER KC AND CARMICHAEL J. (1993). Association between expression of activated 72-kilodalton gelatinase and tumor spread in non-small-cell lung carcinoma. *J. Natl Cancer Inst.*, **85**, 574–578.

CLARK IM, POWELL LK AND CAWSTON TE. (1994). Tissue inhibitor of metalloproteinases (TIMP-1) stimulates the secretion of collagenase from human skin fibroblasts. *Biochem. Biophys. Res. Commun.*, **203**, 874–880.

DAVIES B, MILES DW, HAPPERFIELD LC, NAYLOR MS, BOBROW LG, RUBENS RD AND BALKWILL FR. (1993*a*). Activity of type IV collagenases in benign and malignant breast disease. *Br. J. Cancer*, **67**, 1126–1131.

DAVIES B, WAXMAN J, WASAN H, ABEL P, WILLIAMS G, KRAUSZ T, NEAL D, THOMAS D, HANBY A AND BALKWILL F. (1993*b*). Levels of matrix metalloproteases in bladder cancer correlate with tumor grade and invasion. *Cancer Res.*, **53**, 5365–5369.

DEAN DD, SCHWARTZ ZV, MUNIZ OE, GOMEZ R, SWAIN LD, HOWELL DS AND BOYAN BD. (1992). Matrix vesicles contain metalloproteinases that degrade proteoglycans. *Bone Miner.*, **17**, 172–176.

DECLERCK YA. (1988). Purification and characterization of a collagenase inhibitor produced by bovine vascular smooth muscle cells. *Arch. Biochem. Biophys.*, **265**, 28–37.

DOCHERTY AJP, O'CONNELL J, CRABBE T, ANGAL S AND MURPHY G. (1992). The matrix metelloproteinases and their inhibitors. *Trends Biotech.*, **10**, 200–207.

ENNIS BW AND MATRISIAN LM. (1994). Matrix degrading metalloproteinases. *J. Neuro-Oncol.*, **18**, 105–109.

FEINBERG AP AND VOGELSTEIN B. (1983). A technique for radiolabeling DNA restriction endonuclease fragments to high specific activity. *Anal. Biochem.*, **132**, 6–12.

GASSON JC, GOLDE DW, KAUFMAN CA, WESTBROOK CA, HEWICK RM, KAUFMAN RJ, WONG GG, TEMPLE PA, LEARY AC, BROWN EL, ORR EC AND CLARK SC. (1985). Molecular characterization and expression of the gene encoding human erythoid-potentiating activity. *Nature*, **315**, 768–771.

GAVRILOVIC J, HEMBRY RM, REYNOLDS JJ AND MURPHY G. (1987). Tissue inhibitor of metalloproteinases (TIMP) regulates extracellular type I collagen degradation by chondrocytes and endothelial cells. *J. Cell Sci.*, **87**, 357–362.

GUILLEM JG, LEVY MF, HSIEH LL, JOHNSON MD, LOGERFO P, FORDE KA AND WEINSTEIN IB. (1990). Increased levels of phorbin, c-myc, and ornithine decarboxylase RNAs in human colon cancer. *Mol. Carcinogen.*, **3**, 68–74.

HAYAKAWA T, YAMASHITA K, TANZAWA K, UCHIJIMA E AND IWATA K. (1992). Growth-promoting activity of tissue inhibitor of metalloproteinases-1 (TIMP-1) for a wide range of cells. A possible new growth factor in serum. *FEBS Lett.*, **298**, 29–32.

HERRON MS, BANDA MJ, CLARK EJ, GAVRILOVIC J AND WERB Z. (1988). Secretion of metalloproteinases by stimulated capillary endothelial cells. II. Expression of collagenase and stromelysin activities is regulated by endogenous inhibitors. *J. Biol. Chem.*, **261**, 2814–2818.

HEWITT RE, LEACH IH, POWE DG, CLARK IM, CAWSTON TE AND TURNER DR. (1991). Distribution of collagenase and tissue inhibitor of metalloproteinases (TIMP) in colorectal tumours. *Int. J. Cancer*, **49**, 666–672.

HIBBS MS, HASTY KA, SEYER JM, KANG AH AND MAINARDI CI. (1985). Biochemical and immunological characterization of the secreted forms of human neutrophil gelatinase. *J. Biol. Chem.*, **260**, 2493–2500.

HIBBS MS, HOIDAL JR AND KANG AH. (1987). Expression of a metalloproteinase that degrades native type IV collagen and denatured collagens by cultured human alveolar macrophages. *J. Clin. Invest.*, **80**, 1644–1650.

KHOKHA R, WATERHOUSE P, YAGEL S, LALA PK, OVERALL CM, NORTON G AND DENHARDT DT. (1989). Antisense RNA-induced reduction in murine TIMP levels confers oncogenicity of swiss 3T3 cells. *Science*, **243**, 947–950.

LEVY AT, CIOCE V, SOBEL ME, GARBISA S, GRIGIONI WF, LIOTTA LA AND STETLER-STEVENSON WG. (1991). Increased expression of the Mr 72,000 type IV collagenase in human colonic adenocarcinoma. *Cancer Res.*, **51**, 439–444.

LIOTTA LA, TRYGGVASON K, GARBISA S, HART I, FOLTZ CM AND SHAFIE S. (1980). Metastatic potential correlates with enzymatic degradation of basement membrane collagen. *Nature*, **284**, 67–68.

LIOTTA LA AND STETLER-STEVENSON WG. (1991). Tumour invasion and metastasis: an imbalance of positive and negative regulation. *Cancer Res.*, **51**, 5054s–5059s.

LU XQ, LEVY M, WEINSTEIN IB AND SANTELLA RM. (1991). Immunological quantitation of levels of tissue inhibitor of metalloproteinase-1 in human colon cancer. *Cancer Res.*, **51**, 6231–6235.

MURPHY G, CAWSTON T AND REYNOLDS J. (1981). An inhibitor of collagenase from human amniotic fluid. Purification, characterization and action on metalloproteinases. *Biochemistry*, **195**, 167–170.

MURPHY G, REYNOLDS JJ AND HEMBRY RM. (1989). Metalloproteinases and cancer invasion and metastasis. *Int. J. Cancer*, **44**, 757–760.

NAKAJIMA M, WELCH DR, BELLONI PN AND NICOLSON GL. (1987). Degradation of basement membrane type IV collagen and lung subendothelial matrix by rat mammary adenocarcinoma cell clones of differing metastatic potentials. *Cancer Res.*, **47**, 4869–4876.

NEWELL KJ, WITTY JP, RODGERS WH AND MATRISIAN LM. (1994). Expression and localization of matrix-degrading metalloproteinases during colorectal tumorigenesis. *Mol. Carcinogen.*, **10**, 199–206.

PAVLOFF N, STASKUS PW, KISHNANI NS AND HAWKES SP. (1992). A new inhibitor of metalloproteinases from chicken: ChIMP-3. A third member of the TIMP family. *J. Biol. Chem.*, **267**, 17321–17326.

PULFORD KAF, RIGNEY EM, MICKLEM KJ, JONES M, STROSS WP, GATTER KC AND MASON DR. (1989). KP1: a new monoclonal antibody that detects a monocyte macrophage associated antigen in routinely processed tissue sections. *J. Clin. Pathol.*, **42**, 414–421.

PYKE C, RALFKIAER E, HUHTALA P, HURSKAINEN T, DANO K AND TRYGGVASON K. (1992). Localization of messenger RNA for Mr 72,000 and 92,000 type IV collagenases in human skin cancers by in situ hybridization. *Cancer Res.*, **52**, 1336–1341.

PYKE C, RALKIAER E, TRYGGVASON K AND DANO K. (1993). Messenger RNA for two type IV collagenases is located in stromal cells in human colon cancer. *Am. J. Pathol.*, **142**, 359–365.

SAARIALHO-KERE UK, CHANG ES, WELGUS HG AND PARKS WC. (1993). Expression of interstitial collagenase, 92-kDa gelatinase, and tissue inhibitor of metalloproteinases-1 in granuloma annulare and necrobiosis lipoidica diabeticorum. *J. Invest. Dermatol.*, **100**, 335–342.

SATO H, KIDA Y, MAI M, ENDO Y, SASAKI T, TANAKA J AND SEIKI M. (1992). Expression of genes encoding type IV collagen-degrading metalloproteinases and tissue inhibitors of metalloproteinases in various human tumor cells. *Oncogene*, **7**, 77–83.

SCHULTZ RM, SILBERMAN S, PERSKY B, BAJKOWSKI AS AND CARMICHAEL DF. (1988). Inhibition by human recombinant tissue inhibitor of metalloproteinases of human amnion invasion and lung colonization by murine B16-F10 melanoma cells. *Cancer Res.*, **48**, 5539–5545.

STETLER-STEVENSON WG, KRUTZSCH HC AND LIOTTA LA. (1989). Tissue inhibitor of metalloproteinase (TIMP-2). A new member of the metalloproteinase inhibitor family. *J. Biol. Chem.*, **264**, 17374–17378.

STETLER-STEVENSON WG, BERSCH N AND GOLDE DW. (1992). Tissue inhibitor of metalloproteinase-2 (TIMP-2) has erythroid-potentiating activity. *FEBS Lett.*, **296**, 231–234.

STRICKLIN GP AND WELGUS HG. (1983). Human skin fibroblast collagenase inhibitor. *J. Biol. Chem.*, **258**, 12252–12258.

URA H, BONFIL RD, REICH R, REDDEL R, PFEIFER A, HARRIS CC AND KLEIN-SZANTO AJ. (1989). Expression of type IV collagenase and procollagen genes and its correlation with the tumorigenic, invasive, and metastatic abilities of oncogene-transformed human bronchial epithelial cells. *Cancer Res.*, **49**, 4615–4621.

URBANSKI SJ, EDWARDS DR, MAITLAND A, LECO KJ, WATSON A AND KOSSAKOWSKA AE. (1992). Expression of metalloproteinases and their inhibitors in primary pulmonary carcinomas. *Br. J. Cancer*, **66**, 1188–1194.

WERB Z AND ALEXANDER CM. (1993). Proteinase and matrix degradation. In *Textbook of Rheumatology*, Kelley WN, Harris Jr ED, Ruddy S and Sledge CB. (eds) pp. 248–268. W.B. Saunders: Philadelphia.

WILHELM SM, COLLIER IE, MARMER BL, EISEN AZ, GRANT GA AND GOLDBERG GI. (1989). SV40-transformed human lung fibroblasts secrete a 92-kDa type IV collagenase which is identical to that secreted by normal human macrophages. *J. Biol. Chem.*, **264**, 17213–17221.

WOESSNER JR JF. (1994). The family of matrix metalloproteinases. *Ann. NY Acad. Sci.*, **732**, 11–21.

ZENG ZS, COHEN AM, STETLER-STEVENSON W AND GUILLEM JG. (1993). Parallel rise in expression of 92 Kd type IV collagenase and tissue inhibitor of metalloproteinase-1 (TIMP-1) in human colorectal cancer and liver metastases. *Proc. Annu. Meeting Am. Assoc. Cancer Res.*, **34**, 79.

ZENG, ZS, HSU S, ZHANG ZF, COHEN AM, ENKER WE, TURNBULL AA AND GUILLEM JG. (1994*a*). High level of Nm23-H1 gene expression is associated with local colorectal cancer progression not with metastases. *Br. J. Cancer*, **70**, 1025–1030.

ZENG ZS, SARKIS AS, ZHANG ZF, KLIMSTRA DS, CHARYTONOWICZ E, GUILLEM JG, CORDON-CARDO C AND COHEN AM. (1994*b*). p53 nuclear overexpression: an independent predictor of survival in lymph node-positive colorectal cancer patients. *J. Clin. Oncol.*, **12**, 2043–2050.

ZENG ZS, COHEN AM, ZHANG ZF, STETLER-STEVENSON W AND GUILLEM JG. (1995). Elevated tissue inhibitor of metalloproteinase-1 (TIMP-1) RNA in colorectal cancer stroma correlates with lymph node and distant metastases. *Clin. Cancer Res.*, (in press).


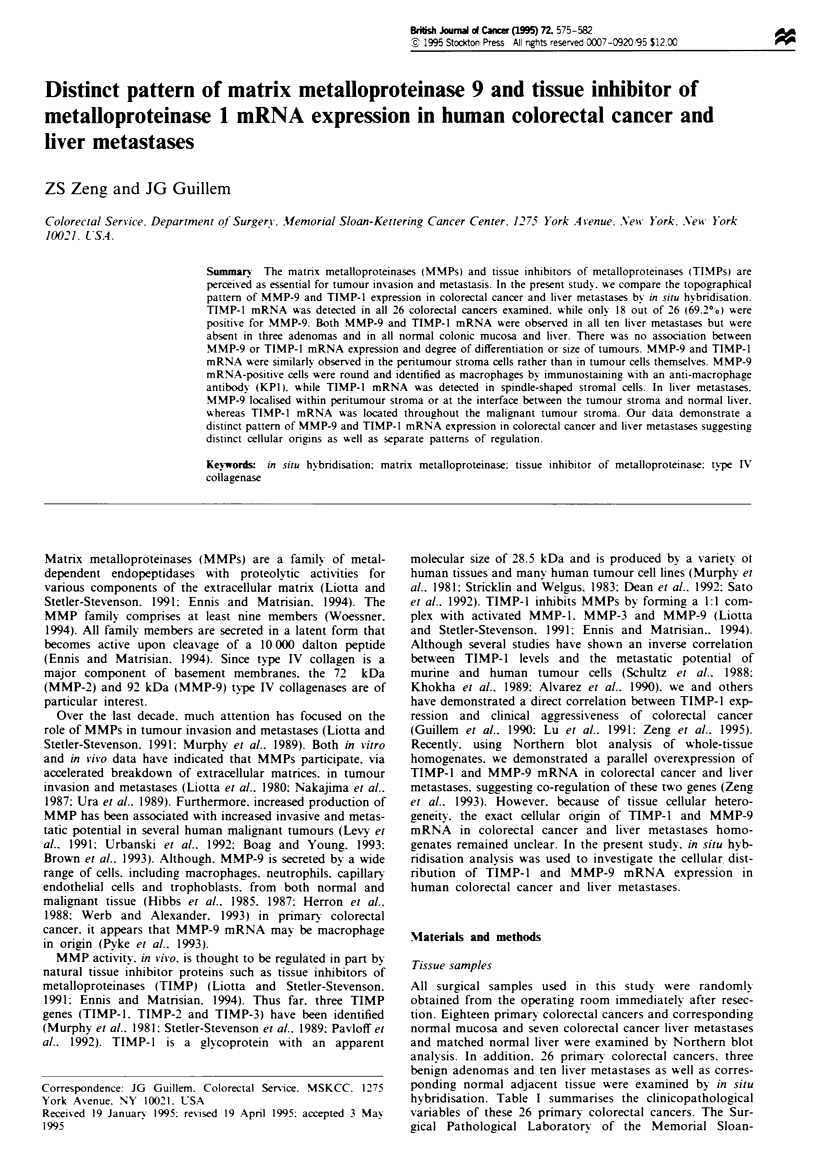

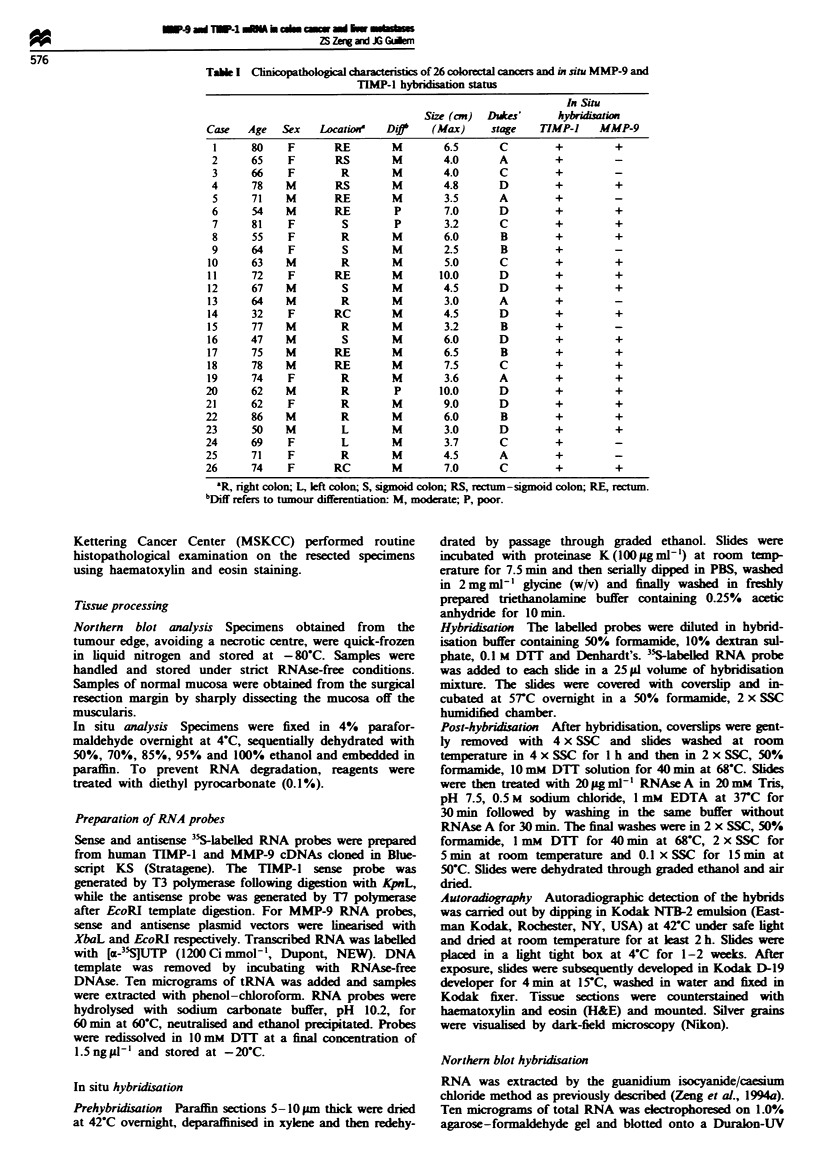

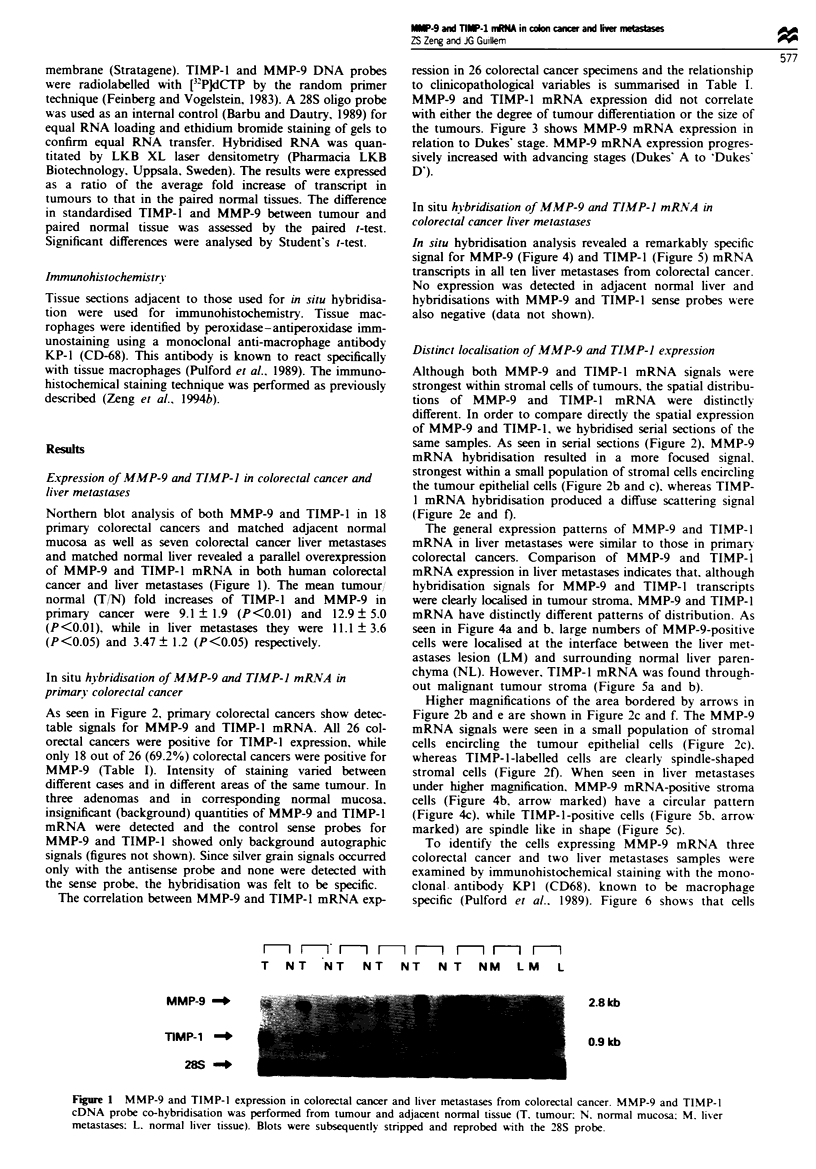

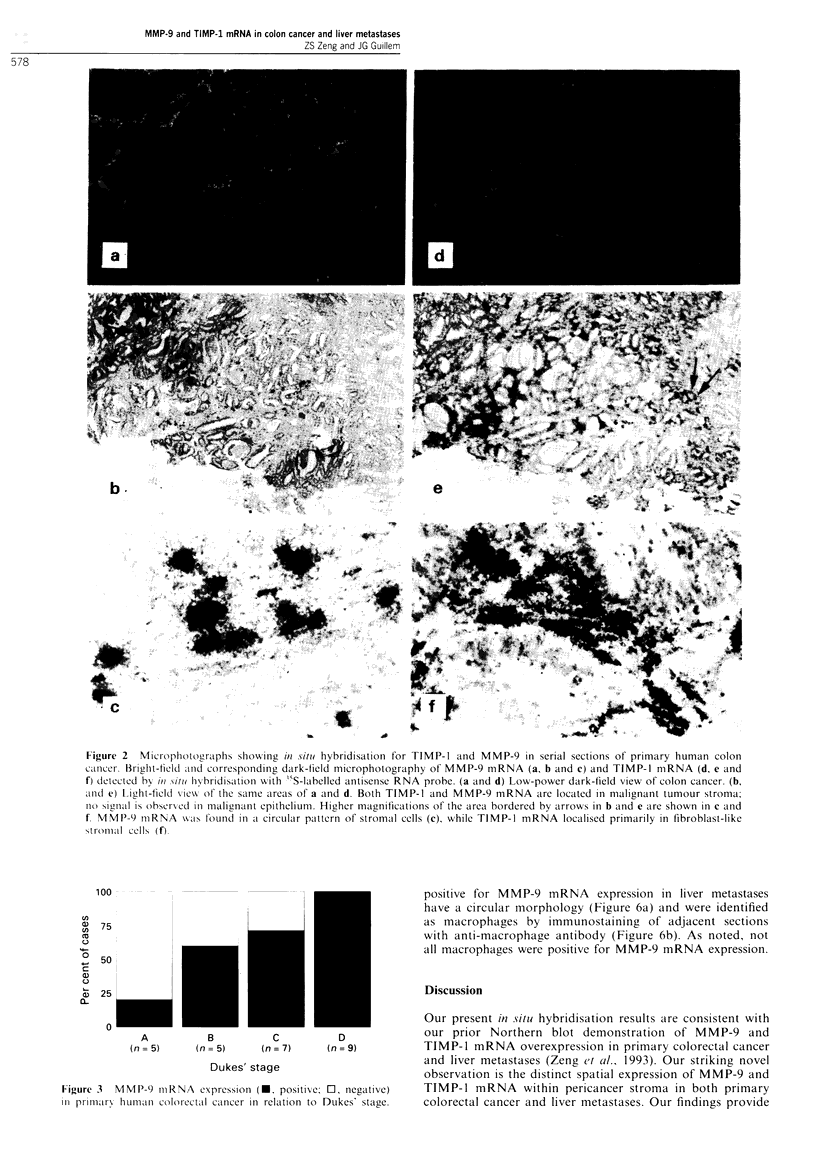

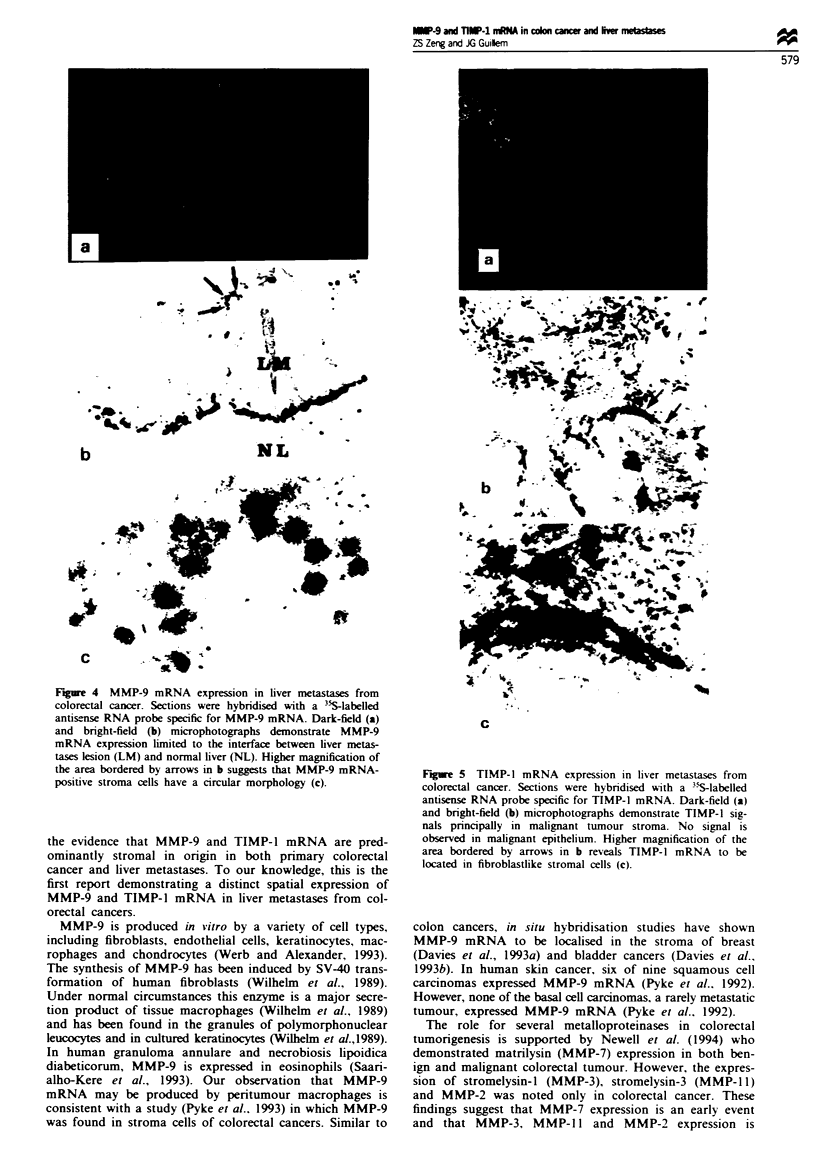

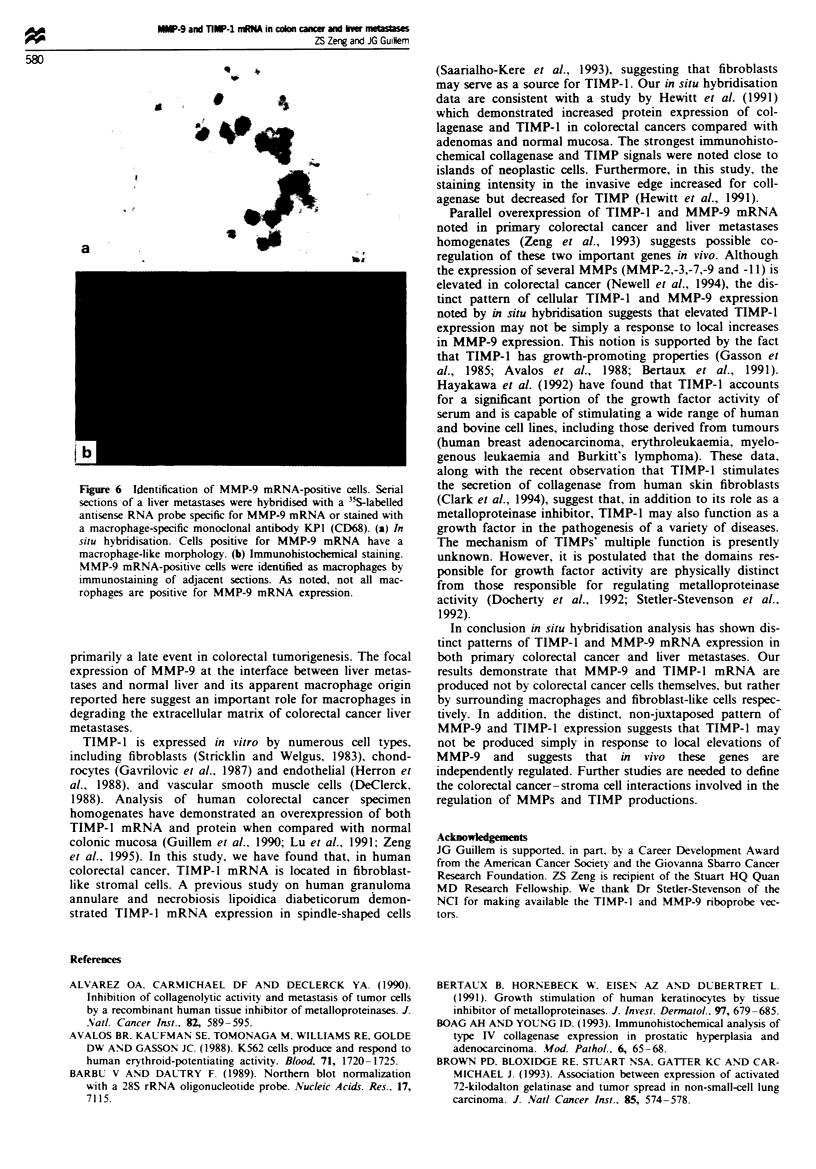

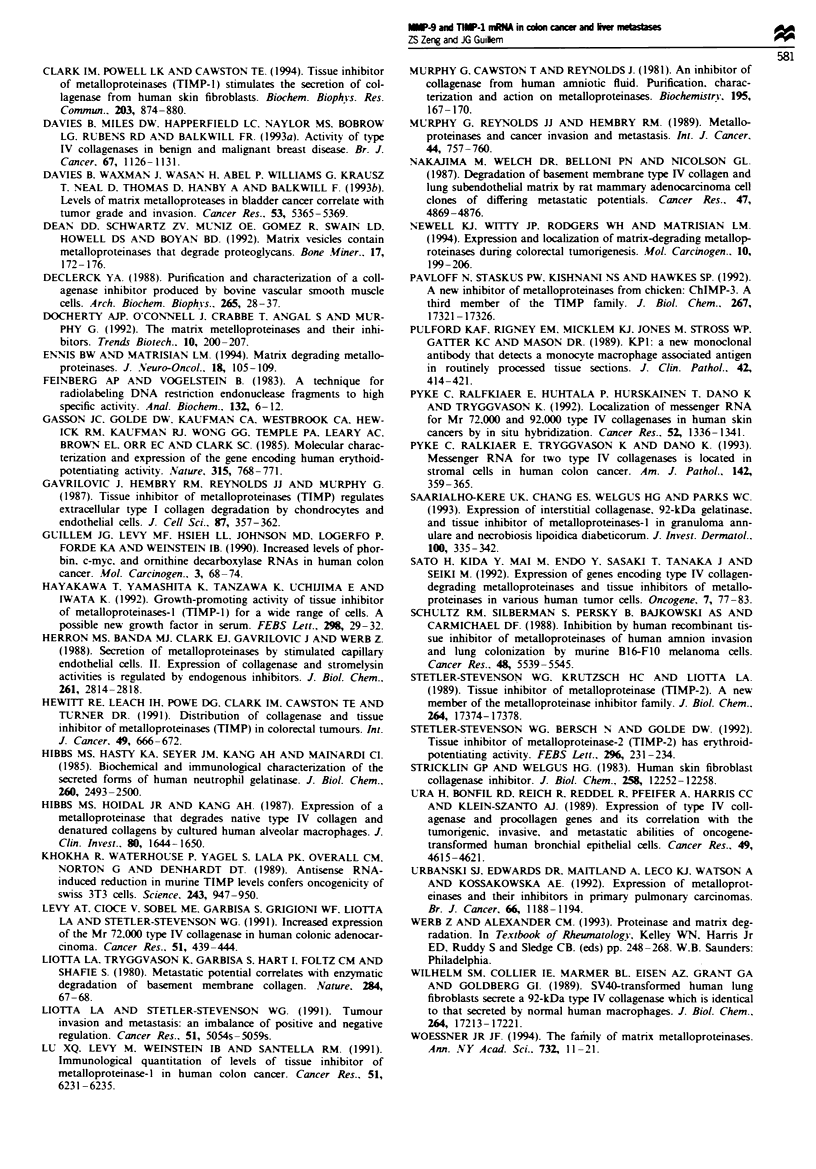

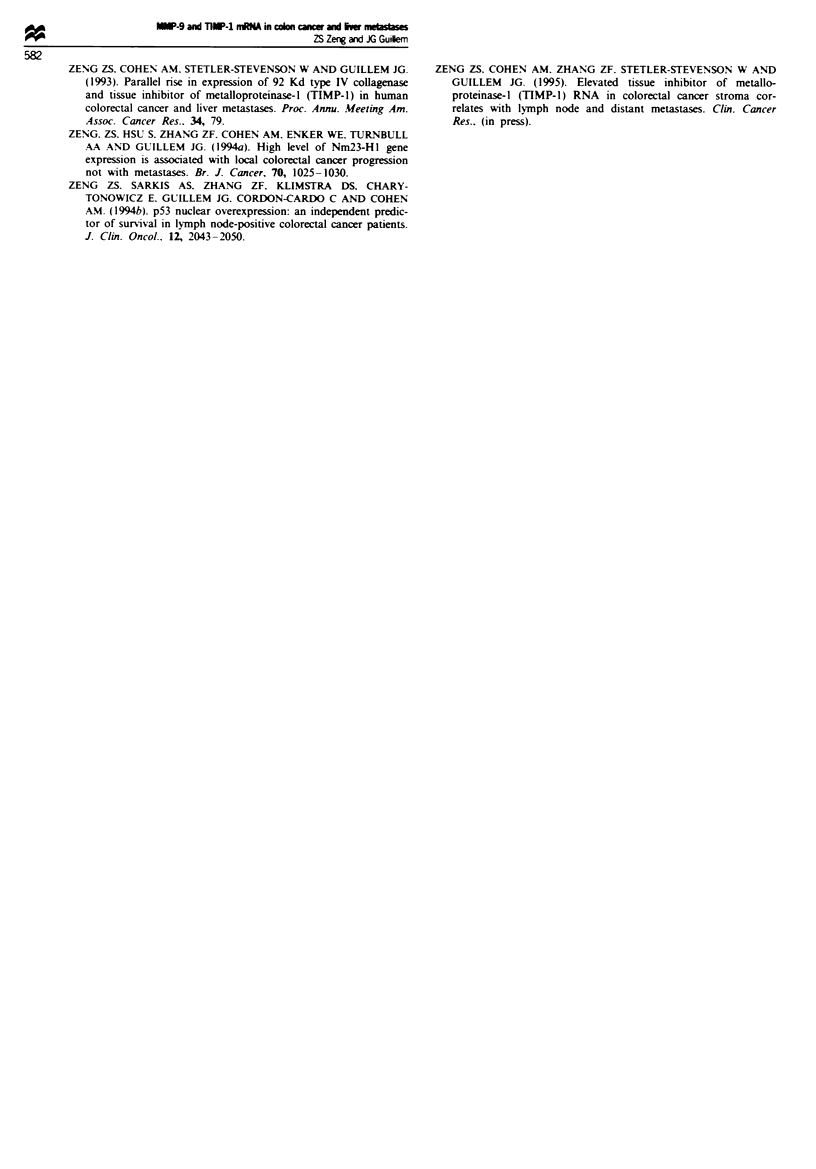


## References

[OCR_00895] Alvarez O. A., Carmichael D. F., DeClerck Y. A. (1990). Inhibition of collagenolytic activity and metastasis of tumor cells by a recombinant human tissue inhibitor of metalloproteinases.. J Natl Cancer Inst.

[OCR_00901] Avalos B. R., Kaufman S. E., Tomonaga M., Williams R. E., Golde D. W., Gasson J. C. (1988). K562 cells produce and respond to human erythroid-potentiating activity.. Blood.

[OCR_00906] Barbu V., Dautry F. (1989). Northern blot normalization with a 28S rRNA oligonucleotide probe.. Nucleic Acids Res.

[OCR_00913] Bertaux B., Hornebeck W., Eisen A. Z., Dubertret L. (1991). Growth stimulation of human keratinocytes by tissue inhibitor of metalloproteinases.. J Invest Dermatol.

[OCR_00917] Boag A. H., Young I. D. (1993). Immunohistochemical analysis of type IV collagenase expression in prostatic hyperplasia and adenocarcinoma.. Mod Pathol.

[OCR_00922] Brown P. D., Bloxidge R. E., Stuart N. S., Gatter K. C., Carmichael J. (1993). Association between expression of activated 72-kilodalton gelatinase and tumor spread in non-small-cell lung carcinoma.. J Natl Cancer Inst.

[OCR_00934] Clark I. M., Powell L. K., Cawston T. E. (1994). Tissue inhibitor of metalloproteinases (TIMP-1) stimulates the secretion of collagenase from human skin fibroblasts.. Biochem Biophys Res Commun.

[OCR_00940] Davies B., Miles D. W., Happerfield L. C., Naylor M. S., Bobrow L. G., Rubens R. D., Balkwill F. R. (1993). Activity of type IV collagenases in benign and malignant breast disease.. Br J Cancer.

[OCR_00947] Davies B., Waxman J., Wasan H., Abel P., Williams G., Krausz T., Neal D., Thomas D., Hanby A., Balkwill F. (1993). Levels of matrix metalloproteases in bladder cancer correlate with tumor grade and invasion.. Cancer Res.

[OCR_00956] DeClerck Y. A. (1988). Purification and characterization of a collagenase inhibitor produced by bovine vascular smooth muscle cells.. Arch Biochem Biophys.

[OCR_00950] Dean D. D., Schwartz Z. V., Muniz O. E., Gomez R., Swain L. D., Howell D. S., Boyan B. D. (1992). Matrix vesicles contain metalloproteinases that degrade proteoglycans.. Bone Miner.

[OCR_00961] Docherty A. J., O'Connell J., Crabbe T., Angal S., Murphy G. (1992). The matrix metalloproteinases and their natural inhibitors: prospects for treating degenerative tissue diseases.. Trends Biotechnol.

[OCR_00966] Ennis B. W., Matrisian L. M. (1994). Matrix degrading metalloproteinases.. J Neurooncol.

[OCR_00978] Gasson J. C., Golde D. W., Kaufman S. E., Westbrook C. A., Hewick R. M., Kaufman R. J., Wong G. G., Temple P. A., Leary A. C., Brown E. L. Molecular characterization and expression of the gene encoding human erythroid-potentiating activity.. Nature.

[OCR_00982] Gavrilovic J., Hembry R. M., Reynolds J. J., Murphy G. (1987). Tissue inhibitor of metalloproteinases (TIMP) regulates extracellular type I collagen degradation by chondrocytes and endothelial cells.. J Cell Sci.

[OCR_00988] Guillem J. G., Levy M. F., Hsieh L. L., Johnson M. D., LoGerfo P., Forde K. A., Weinstein I. B. (1990). Increased levels of phorbin, c-myc, and ornithine decarboxylase RNAs in human colon cancer.. Mol Carcinog.

[OCR_00996] Hayakawa T., Yamashita K., Tanzawa K., Uchijima E., Iwata K. (1992). Growth-promoting activity of tissue inhibitor of metalloproteinases-1 (TIMP-1) for a wide range of cells. A possible new growth factor in serum.. FEBS Lett.

[OCR_01001] Herron G. S., Banda M. J., Clark E. J., Gavrilovic J., Werb Z. (1986). Secretion of metalloproteinases by stimulated capillary endothelial cells. II. Expression of collagenase and stromelysin activities is regulated by endogenous inhibitors.. J Biol Chem.

[OCR_01008] Hewitt R. E., Leach I. H., Powe D. G., Clark I. M., Cawston T. E., Turner D. R. (1991). Distribution of collagenase and tissue inhibitor of metalloproteinases (TIMP) in colorectal tumours.. Int J Cancer.

[OCR_01014] Hibbs M. S., Hasty K. A., Seyer J. M., Kang A. H., Mainardi C. L. (1985). Biochemical and immunological characterization of the secreted forms of human neutrophil gelatinase.. J Biol Chem.

[OCR_01020] Hibbs M. S., Hoidal J. R., Kang A. H. (1987). Expression of a metalloproteinase that degrades native type V collagen and denatured collagens by cultured human alveolar macrophages.. J Clin Invest.

[OCR_01024] Khokha R., Waterhouse P., Yagel S., Lala P. K., Overall C. M., Norton G., Denhardt D. T. (1989). Antisense RNA-induced reduction in murine TIMP levels confers oncogenicity on Swiss 3T3 cells.. Science.

[OCR_01030] Levy A. T., Cioce V., Sobel M. E., Garbisa S., Grigioni W. F., Liotta L. A., Stetler-Stevenson W. G. (1991). Increased expression of the Mr 72,000 type IV collagenase in human colonic adenocarcinoma.. Cancer Res.

[OCR_01044] Liotta L. A., Stetler-Stevenson W. G. (1991). Tumor invasion and metastasis: an imbalance of positive and negative regulation.. Cancer Res.

[OCR_01038] Liotta L. A., Tryggvason K., Garbisa S., Hart I., Foltz C. M., Shafie S. (1980). Metastatic potential correlates with enzymatic degradation of basement membrane collagen.. Nature.

[OCR_01049] Lu X. Q., Levy M., Weinstein I. B., Santella R. M. (1991). Immunological quantitation of levels of tissue inhibitor of metalloproteinase-1 in human colon cancer.. Cancer Res.

[OCR_01055] Murphy G., Cawston T. E., Reynolds J. J. (1981). An inhibitor of collagenase from human amniotic fluid. Purification, characterization and action on metalloproteinases.. Biochem J.

[OCR_01061] Murphy G., Reynolds J. J., Hembry R. M. (1989). Metalloproteinases and cancer invasion and metastasis.. Int J Cancer.

[OCR_01066] Nakajima M., Welch D. R., Belloni P. N., Nicolson G. L. (1987). Degradation of basement membrane type IV collagen and lung subendothelial matrix by rat mammary adenocarcinoma cell clones of differing metastatic potentials.. Cancer Res.

[OCR_01073] Newell K. J., Witty J. P., Rodgers W. H., Matrisian L. M. (1994). Expression and localization of matrix-degrading metalloproteinases during colorectal tumorigenesis.. Mol Carcinog.

[OCR_01079] Pavloff N., Staskus P. W., Kishnani N. S., Hawkes S. P. (1992). A new inhibitor of metalloproteinases from chicken: ChIMP-3. A third member of the TIMP family.. J Biol Chem.

[OCR_01086] Pulford K. A., Rigney E. M., Micklem K. J., Jones M., Stross W. P., Gatter K. C., Mason D. Y. (1989). KP1: a new monoclonal antibody that detects a monocyte/macrophage associated antigen in routinely processed tissue sections.. J Clin Pathol.

[OCR_01093] Pyke C., Ralfkiaer E., Huhtala P., Hurskainen T., Danø K., Tryggvason K. (1992). Localization of messenger RNA for Mr 72,000 and 92,000 type IV collagenases in human skin cancers by in situ hybridization.. Cancer Res.

[OCR_01098] Pyke C., Ralfkiaer E., Tryggvason K., Danø K. (1993). Messenger RNA for two type IV collagenases is located in stromal cells in human colon cancer.. Am J Pathol.

[OCR_01104] Saarialho-Kere U. K., Chang E. S., Welgus H. G., Parks W. C. (1993). Expression of interstitial collagenase, 92-kDa gelatinase, and tissue inhibitor of metalloproteinases-1 in granuloma annulare and necrobiosis lipoidica diabeticorum.. J Invest Dermatol.

[OCR_01111] Sato H., Kida Y., Mai M., Endo Y., Sasaki T., Tanaka J., Seiki M. (1992). Expression of genes encoding type IV collagen-degrading metalloproteinases and tissue inhibitors of metalloproteinases in various human tumor cells.. Oncogene.

[OCR_01116] Schultz R. M., Silberman S., Persky B., Bajkowski A. S., Carmichael D. F. (1988). Inhibition by human recombinant tissue inhibitor of metalloproteinases of human amnion invasion and lung colonization by murine B16-F10 melanoma cells.. Cancer Res.

[OCR_01129] Stetler-Stevenson W. G., Bersch N., Golde D. W. (1992). Tissue inhibitor of metalloproteinase-2 (TIMP-2) has erythroid-potentiating activity.. FEBS Lett.

[OCR_01123] Stetler-Stevenson W. G., Krutzsch H. C., Liotta L. A. (1989). Tissue inhibitor of metalloproteinase (TIMP-2). A new member of the metalloproteinase inhibitor family.. J Biol Chem.

[OCR_01134] Stricklin G. P., Welgus H. G. (1983). Human skin fibroblast collagenase inhibitor. Purification and biochemical characterization.. J Biol Chem.

[OCR_01136] Ura H., Bonfil R. D., Reich R., Reddel R., Pfeifer A., Harris C. C., Klein-Szanto A. J. (1989). Expression of type IV collagenase and procollagen genes and its correlation with the tumorigenic, invasive, and metastatic abilities of oncogene-transformed human bronchial epithelial cells.. Cancer Res.

[OCR_01147] Urbanski S. J., Edwards D. R., Maitland A., Leco K. J., Watson A., Kossakowska A. E. (1992). Expression of metalloproteinases and their inhibitors in primary pulmonary carcinomas.. Br J Cancer.

[OCR_01156] Wilhelm S. M., Collier I. E., Marmer B. L., Eisen A. Z., Grant G. A., Goldberg G. I. (1989). SV40-transformed human lung fibroblasts secrete a 92-kDa type IV collagenase which is identical to that secreted by normal human macrophages.. J Biol Chem.

[OCR_01163] Woessner J. F. (1994). The family of matrix metalloproteinases.. Ann N Y Acad Sci.

[OCR_01182] Zeng Z. S., Hsu S., Zhang Z. F., Cohen A. M., Enker W. E., Turnbull A. A., Guillem J. G. (1994). High level of Nm23-H1 gene expression is associated with local colorectal cancer progression not with metastases.. Br J Cancer.

[OCR_01185] Zeng Z. S., Sarkis A. S., Zhang Z. F., Klimstra D. S., Charytonowicz E., Guillem J. G., Cordon-Cardo C., Cohen A. M. (1994). p53 nuclear overexpression: an independent predictor of survival in lymph node--positive colorectal cancer patients.. J Clin Oncol.

